# Robust Optimization Scheme for Inverse Method for Crystal Plasticity Model Parametrization

**DOI:** 10.3390/ma13030735

**Published:** 2020-02-06

**Authors:** Mahdieh Shahmardani, Napat Vajragupta, Alexander Hartmaier

**Affiliations:** 1 Interdisciplinary Centre for Advanced Materials Simulation (ICAMS), Ruhr-Universität Bochum, Universitätsstr. 150, 44801 Bochum, Germany; napat.vajragupta@rub.de (N.V.); alexander.hartmaier@rub.de (A.H.); 2 Department of Mechanical Engineering, Faculty of Engineering, Blekinge Institute of Technology, 371 79 Karlskrona, Sweden

**Keywords:** nanoindentation test, inverse analysis, trust-region-reflective algorithm, nonlocal crystal plasticity, geometry necessary dislocation, BCC material

## Abstract

A bottom-up material modeling based on a nonlocal crystal plasticity model requires information of a large set of physical and phenomenological parameters. Because of the many material parameters, it is inherently difficult to determine the nonlocal crystal plasticity parameters. Therefore, a robust method is proposed to parameterize the nonlocal crystal plasticity model of a body-centered cubic (BCC) material by combining a nanoindentation test and inverse analysis. Nanoindentation tests returned the load–displacement curve and surface imprint of the considered sample. The inverse analysis is developed based on trust-region-reflective algorithm, which is the most robust optimization algorithm for the considered non-convex problem. The discrepancy function is defined to minimize both the load–displacement curves and the surface topologies of the considered material under applying varied indentation forces obtained from numerical models and experimental output. The numerical model results based on the identified material properties show good agreement with the experimental output. Finally, a sensitivity analysis performed changing the nonlocal crystal plasticity parameters in a predefined range emphasized that the geometrical factor has the most significant influence on the load–displacement curve and surface imprint parameters.

## 1. Introduction

Nanoindentation is a technique for testing the mechanical properties of materials in the nanometer scale utilizing instruments with high precision. In the region underneath the indenter, nanoindentation results in complex stress distributions that produce non-uniform strain [1,2]. Nanoindentation can be used for various applications including mineral analysis, thin films testing, scratch testing, and structural characterization of weld materials [3,4,5,6].

To distinguish the main principle of the mechanical properties of materials, it is essential to investigate their deformation mechanism. However, describing the detailed deformation mechanism occurring in a material is inherently difficult [7,8,9]. To understand the deformation mechanism at the grain scale, the micromechanical modeling approach is applied [10]. The micromechanical model uses microstructural features in combination with a material model to reflect the behavior of crystalline materials. Among existing material models, the crystal plasticity finite element (CPFE) simulation can give a rather comprehensive understanding of the nanoindentation process.

Because of an advancement in computational power, accurate simulation of the plastic deformation process of different crystalline materials has been carried out by the CPFE method successfully in recent decades [11,12,13,14]. Several researchers have studied the plastic anisotropy behavior of various materials during nanoindentation by numerical simulation [15,16,17,18,19], in which the CPFE models were usually adopted.

Although, in some of the proposed crystal plasticity theories, the effect of deformation gradients was neglected, size effects are crucial in some applications based on experimental results, such as in bending of polycrystalline nickel [20], micro-bending experiments of single crystal copper and single crystal aluminum [21,22], and twisting of polycrystalline copper [23]. To consider the influence of the deformation gradient, advanced nonlocal constitutive models have been proposed. Most of these constitutive models are derived based on the concept of the geometrically necessary dislocation (GND) density tensor [24,25,26]. These nonlocal crystal plasticity models consist of numerous physical and phenomenological parameters, and characterizing these parameters directly from experimental tests is inherently difficult and therefore makes it necessary to use inverse analysis technique to obtain these parameters.

Due to its efficiency compared to performing standard tests, the inverse analysis of nanoindentation data for predicting and measuring mechanical properties has attracted increasing interest in the scientific community for different material applications [27,28,29,30,31,32,33,34,35]. To precisely evaluate the contact area or for inverse analysis purposes, imprint profiles from indentation tests have been considered for ceramics, metals, and, recently, micro-electro-mechanical systems (MEMS) devices [36,37,38,39,40].

The goal of inverse indentation problem is to identify the unknown mechanical properties of a material obtained from experimental indentation testing including the load–depth record and the surface imprint. There are three main inverse analysis techniques that can be employed to extract mechanical properties of materials from instrumented indentation experimental data: the representative stress–strain method [41,42,43,44,45,46,47,48], the iterative finite element analysis [27,28,29,30,32,33], and artificial neural networks [49,50,51,52]. By using the representative stress–strain method, one must define functions for mapping load–displacement curves to stress–strain curves, which is rather complicated for the case of nanoindentation tests. On the other hand, the inverse method using the iterative finite element analysis resembles the nanoindentation test conditions and its complexity refers only to the material behavior defined in the model. 

Inverse analysis by iterative finite element simulations requires two main prerequisites: precision and uniqueness. The former means that the model is sufficiently accurate and representative of the real experiment. The latter assumes that there is only one set of material parameters for which the simulation produces a load–depth curve that replicates the experimental load–depth curve. If this were not the case, it would be possible for materials with two different properties to generate the same load–depth trace. As a result, if this were true, it would not be possible to uniquely identify the behavior of the indented material through inverse analysis. The issue of uniqueness has proved to be a non-trivial subject and it has been studied by several authors [53,54,55,56,57,58]. This study focused on the inverse analysis technique by iterative finite element simulations because of its simplicity for modeling of the nanoindentation test.

The solution of inverse problems relies upon classical optimization techniques. The proper technique depends on the type of function to be optimized and constraints between parameters. Trust region algorithms are a class of relatively new algorithms [59,60]. The trust region approach is strongly associated with an approximation. In fact, most line search algorithms can find the approximate models using search directions. However, in a trust region algorithm, the discrepancy function is approximated from a nearby region of the current iteration. This seems reasonable because, for general nonlinear functions, local approximate models (such as linear approximation and quadratic approximation) can only fit the original function locally [61]. Since the trust-region-reflective method is a derivative-based algorithm, the converged solution is obtained with fewer iterations compared to other nonlinear optimization methods. For example, in Bayesian optimization using Gaussian process regression, the objective function is unknown and it does not need to calculate the derivatives and these two features increase number of required iterations in optimization process for reaching the converged solution [62].

CPFE contains several material parameters and uniqueness is an issue for calibration of these parameters. Apart from the uniqueness, robustness of the inverse method is another critical feature for actual applications. To achieve a converged solution, these two criteria are considered when defining discrepancy function and choosing the optimization algorithm. Therefore, in this work, we propose the robust optimization scheme for parameterizing nonlocal crystal plasticity model by fitting both load–displacement curves and surface imprints obtained from nanoindentation tests. In the next section, to approximately solve the inverse problem for a given material, finite element models of the experimental set-up are analyzed. Section 3 contains the parameterization of the material and describes the developed discrepancy function and selected algorithm for solving the inverse problem. Then, for the identification purpose, different sets of relevant material properties are used in the simulations until the simulated load–depth curves as well as the surface imprints match the experimentally measured load–depth curves and surface topologies. The combination of material properties used in the finite element model that results in the simulated load–depth curve and in the surface imprint matching the experimental output is assumed to represent the nonlocal crystal plasticity properties being investigated. Section 4 discusses the influence of some of preselected nonlocal crystal plasticity parameters and the effect of their combination on both load–displacement curve and surface imprint parameters. Section 5 summarizes the knowledge gained from this study.

## 2. Nanoindentation Simulation 

For the identification purpose, a finite element model is developed to simulate the performed nanoindentation tests using a finite element commercial code [63] that implements both material and geometrical nonlinearity.

### 2.1. Numerical Model of Nanoindentation

**Figure 1:**
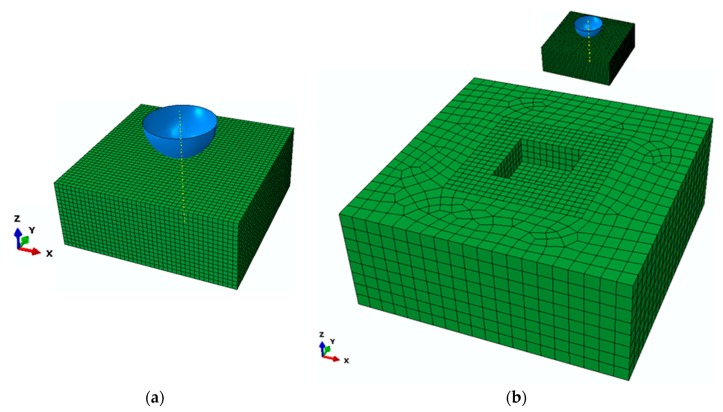
The numerical model of the nanoindentation test: (**a**) with indented solid mesh discretization; and (**b**) with an outer cube to enclose the indented grain.

The nanoindentation model simulating the experimental test conditions [64] is represented in Figure 1a. The indented single grain has 25 μm length, 25 μm width, and 10 μm thickness, which is sufficiently large to cover the occurred plastic zone of the indentation region [64]. Due to the non-symmetric behavior of the material, this numerical model excludes the symmetry and considers the entire experimental set-up.

To accurately calculate of strain gradients and to accommodate a strong deformation field at the contact region, the mesh was refined. Therefore, the indented grain is discretized regularly with eight-node linear brick (C3D8) elements, of which the element size is approximately 0.6 μm and which totally includes 25,600 elements.

A larger cube visualized in Figure 1b was modeled to enclose the indented single grain to support it under applying load by the indenter. The size of the outer cube is 100 × 100 × 40 μm^3^ and discretized with a 20-node quadratic brick element (C3D20), and its behavior is described only in the elastic regime.

Based on the indenter used in the experiment [64], the sphero-conical indenter was modeled as an analytical rigid body because of its high stiffness compared to the specimen with a radius of 5 μm and an angle of 90 degrees. However, because only the spherical part of the indenter was in contact with the indented single grain, we modeled only this part of the indenter. It is assumed that the contact between indenter and grain is frictionless.

### 2.2. Nonlocal Crystal Plasticity Model

The considered material behavior is described by the nonlocal crystal plasticity model as proposed by Ma and Hartmaier [26]. It has been implemented in Abaqus by a user-defined material subroutine (UMAT), which is coupled to the finite element model to simulate the nanoindentation test. Since the described crystal plasticity model in this study follows concepts of fundamental work [65,66,67], we focused only on the details of the non-local formulation and the relevant material parameters. 

With the assumption of the kinematics of deformation, the total deformation gradient tensor, F, is multiplicatively decomposed to

(1) $$F\;=\;F^{e}F^{p}$$

where $F^{e}$ and $F^{p}$ are the elastic and the plastic part of the deformation gradient tensor, respectively. The plastic deformation, $F^{p}$, which consists of an irreversible permanent deformation, evolves as

(2) $$\dot{F}=L^{p}F^{p}$$

where $L^{p}$ is the plastic part of the gradient velocity tensor, and, since in this study dislocation slip is considered as the only deformation process, results in

(3) $$L^{p}=\sum_{\alpha =1}^{N}{\dot{\gamma }}_{\alpha }{\tilde{M}}_{\alpha }$$

where ${\dot{\gamma }}_{\alpha }$ is the slip rate and ${\tilde{M}}_{\alpha }=d_{\alpha }⊗n_{\alpha }$ defines the Schmid tensor for the slip system α, which is defined by the slip direction $d_{\alpha }$ and the slip plane normal $n_{\alpha }$. The symbol ⊗ denotes the dyadic product of two vectors resulting in a second rank tensor. N counts the total number of slip systems.

Th elastic response can be obtained by calculating the second Piola–Kirchhoff stress tensor, $\tilde{S}$, as

(4) $$\tilde{S}=\frac{1}{2}\tilde{C}(F^{e^{T}}F^{e}-I)$$

where $\tilde{C}$ is the stiffness tensor. Then, the Cauchy stress is defined as

(5) $$\sigma =\frac{1}{\det F^{e}}F^{e}\tilde{S}F^{e}{}^{T}$$

The plastic deformation mechanism here is governed by the slip mechanism where dislocations slip in well-designed slip systems.

In the plastic regime, the flow rule and the strain hardening law, are defined as below:

(6) $${\dot{\gamma }}_{\alpha }={\dot{\gamma }}_{0}\;|\frac{\tau_{\alpha }+\tau_{\alpha }^{GNDk}}{{\hat{\tau }}_{\alpha }+{\hat{\tau }}_{\alpha }^{GNDi}}{|}^{p_{1}}\;sign(\tau_{\alpha }+\tau_{\alpha }^{GNDk})$$

(7) $${\dot{\hat{\tau }}}_{\alpha }=\sum_{\beta =1}^{N_{S}}h_{0}\chi_{\alpha \beta }(1-\frac{{\hat{\tau }}_{\alpha }}{{\hat{\tau }}_{sat}}{)}^{p_{2}}|{\dot{\gamma }}_{\beta }|$$

where ${\dot{\gamma }}_{0}$ is the reference shear rate, $p_{1}$ is the inverse value of the strain rate sensitivity, $h_{0}$ is the initial hardening rate, ${\hat{\tau }}_{sat}$ is the saturation slip resistance and $p_{2}$ is a fitting parameter. The initial value of the slip resistance ${\hat{\tau }}_{\alpha }$ is defined as $\tau_{0}$, and $\chi_{\alpha \beta }$ is the cross-hardening matrix between crystallographic mobile dislocations and super GNDs. 

The flow rule described in Equation (6) includes two back stresses, ${\hat{\tau }}_{\alpha }^{GNDk}$ and $\tau_{\alpha }^{GNDk}$, which define the additional hardening caused by GNDs due to strain gradients [26]. This additional hardening can be separated into isotropic (${\hat{\tau }}_{\alpha }^{GNDk}$) and kinematic ($\tau_{\alpha }^{GNDk}$) hardening contributions.

In the case of treating $F^{p}$ as additional degree of freedom (DOF) to consider the nonlocal effect [68,69], it is possible to calculate the dislocation density tensor in the reference configuration as follows:

(8) $${G\;=\;-(F}^{p}\;\times \;\nabla )$$

The net Burgers vector $\bar{b}$ can be determined with the help of the dislocation density tensor, for an arbitrary unit area with a normal vector $\bar{n}$ [70], as

(9) $$\bar{b}\;=\;G\bar{n}$$

According to the continuum mechanical point of view, it is not possible to uniquely define crystallographic GND or even to consider individual dislocation segments; therefore, the approach of super dislocations is followed here to describe the dislocation Burgers vectors and the line directions in average correctly and, hence, to produce a valid approximation to their far reaching stress fields.

Here, the dislocation density tensor is projected to the global Cartesian coordinates of the system, and the geometrically necessary super dislocations are defined uniquely. Then, the far field stress of the crystallographic GND population can be described with good accuracy [26]. In this way, the GND density tensor is separated into nine independent parts

(10) $$\sum_{\alpha =1}^{9}{\bar{\rho }}_{\alpha }{\bar{d}}_{\alpha }⊗{\bar{t}}_{\alpha }=\frac{G}{b}$$

where ${\bar{d}}_{\alpha }$ and ${\bar{t}}_{\alpha }$ are permutations of the Cartesian unit vectors and b is the norm of the Burgers vector. ${\bar{\rho }}_{\alpha }$ is named as super GND density, in which the three first densities belong to screw super GND densities and the last six ones represent the edge super GND densities.

It has been found that the forest GNDs can produce strong cross hardening for crystallographic mobile dislocations [71,72]. Under the condition that it is not possible to find a unique solution for the crystallographic GNDs caused by gradients of $F^{p}$, it is then needed to investigate the additional passing stress [73] for mobile dislocations caused by super GNDs

(11) $${\hat{\tau }}_{\alpha }^{GNDi}c_{1}\mu b\sqrt{\sum_{\beta =1}^{9}\chi_{\alpha \beta }^{GND}|{\bar{\rho }}_{\beta }|}$$

where $c_{1}$ is a geometrical factor or the Taylor hardening coefficient [26] and μ is the shear modulus. 

With the assumption of small elastic strains, the resolved shear stress, $\tau_{\alpha }$, and the back stress, $\tau_{\alpha }^{GND}$, within the intermediate configuration, are written as

(12) $$\tau_{\alpha }=\tilde{S}{\tilde{M}}_{\alpha }$$

(13) $$\tau_{\alpha }^{GNDk}={\tilde{S}}^{GND}{\tilde{M}}_{\alpha }$$

where ${\tilde{S}}^{GND}$ is the internal stress in the intermediate configurations [26].

The described constitutive law is implemented into Abaqus as material behavior of ARMCO iron, and the dislocation slip is considered on the common crystallographic <111> {110} slip systems.

## 3. Parameterization of the Nonlocal Crystal Plasticity Model

The considered material behavior is described by the nonlocal crystal plasticity model formerly defined and by a user-defined material subroutine (UMAT), which is coupled to the finite element model to simulate the nanoindentation test. The indentation model is only simulated for a single grain with a crystal orientation close to <100> and with Bunge Euler angles of ($\varphi_{1}$, $\varphi_{2}$, $\varphi_{3}$) = (33.26, 11.48, 328.99). Although indentation occurs only along a single axis, the resulting stress state underneath the indenter is always multiaxial in nature. Thus, all possible slip systems of BCC crystal are activated and, hence, the plastic anisotropy of the BCC crystal is fully considered in the parameterization of the non-local crystal plasticity model by nanoindentation testing.

In terms of parameterization of the nonlocal crystal plasticity model by an inverse analysis, an optimization algorithm is implemented. In this study, two sets of material parameters were chosen as the initial parameter sets. The first set listed in Table 1 was taken from [74] and yielded a good agreement with experimental results. In this context, the purpose of parameterization is to obtain a parameter set, which is in a better agreement with the experiment. In addition, another parameter set is defined arbitrarily to investigate the feasibility of the optimization algorithm. Because of their pronounced effect on the load–displacement curve and the residual imprint, *c*_1_, *p*_2_, ${\hat{\tau }}_{sat}$, and $\tau_{0}$ were adapted in the optimization process to reflect the behavior of the material under nanoindentation tests.

### 3.1. Objective Function and Optimization Algorithm

The inverse analysis is used to identify material parameters of ARMCO iron based on the experimental results obtained from the nanoindentation tests [64]. The nonlocal crystal plasticity parameters of the specimen will be recovered by minimizing the discrepancy between the performed experiments and the results obtained from the finite element model of the real sample, which depends on the input material properties.

To optimize the nonlocal crystal plasticity parameters, the development of a proper discrepancy function is a main factor. The discrepancy function, f(*z*), is a function of the material parameters (*z*), and, since it consists of nonlocal crystal plasticity parameters, vector *z* has a nonlinear relationship with the material response, which makes the problem become multivariable nonlinear. Therefore, the current optimization process is a nonlinear multivariable unconstrained one.

The dependence of the computed quantities at the parameter vector *z* is implicitly described by using the constitutive relationships adopted inside the finite element model. This dependence makes the goal function f, a non-explicit and typically non-convex function of *z*. Therefore, optimization of nonlocal crystal plasticity parameters is a non-convex problem.

To solve a non-convex problem, the trust region approach is the most suitable one because of its boundedness. Furthermore, trust region algorithms are reliable and robust, since they can be applied to ill-conditioned problems, and they also have very strong convergence properties [56]. In this study, the trust-region-reflective algorithm was hence chosen. For a better understanding of the algorithm, we provide a short description as follows.

Assume that there is an initial guess of the solution of the optimization problem, an approximate model can be constructed near the current point. A solution of the approximate model can be taken as the next iterate point. The region that the approximate model is trusted is called the trust region. The trust region is adjusted from iteration to iteration. If the computations indicate the approximate model fits the original problem well, the trust region can be enlarged. Otherwise, when the approximate model does not match, the trust region should be reduced.

The key contents of a trust region algorithm are how to compute the trust region trial step and how to decide whether a trial step should be accepted. An iteration of a trust region algorithm has the following form. A trust region is available at the beginning. Then, an approximate model is constructed, and it is solved within the trust region, giving a solution s, which is called the trial step. A merit function is chosen (merit function is first two terms of the Taylor approximation of discrepancy function), which is used for updating the next trust region and for choosing the new iterate point.

To use the optimization algorithm available in MATLAB environment [75], we linked MATLAB to the finite element commercial software (Abaqus). Numerical analyses return the counterparts of the quantities measured in the experiment as a function of the parameters, here collected in vector *z*, representing the material properties. Their optimum value is identified by the minimization of a discrepancy function, f(*z*), defined as follows for the present application.

(14) $$f\left(z\right)\;=\;\overset{M}{\underset{k=1}{\sum }}{\left(\frac{D_{mk}^{diag}{-D}_{ck}^{diag}\left(z\right)}{D_{m\;max}^{diag}}\right)}^{2}+\overset{N}{\underset{j=1}{\sum }}{\left(\frac{D_{mj}^{top}{-D}_{cj}^{top}\left(z\right)}{D_{m\;min}^{top}}\right)}^{2}$$

In the above relationship, superscripts diag and top indicate the displacements from load–displacement diagrams and from the surface topologies, while subscripts m and c refer to measured and computed quantities, respectively. In particular, $D_{ck}^{diag}$ and $D_{mk}^{diag}$ in the left side parentheses indicate the displacements on the load–displacement curve from the experimental and the numerical results for the number of *M* points on the load–displacement graph, and their subtraction is normalized by $D_{m\;max}^{diag}$, which is the maximum measured displacement from the experimental test. In the right side parentheses, $D_{cj}^{top}$ and $D_{mj}^{top}$ represent the surface imprints from the experimental and the numerical output for the number of *N* points on the surface topology diagram, while $D_{m\;min}^{top}$ indicates a normalization term, here assumed to coincide with the minimum measured displacement from the surface imprint experienced at the applied load.

**Figure 2:**
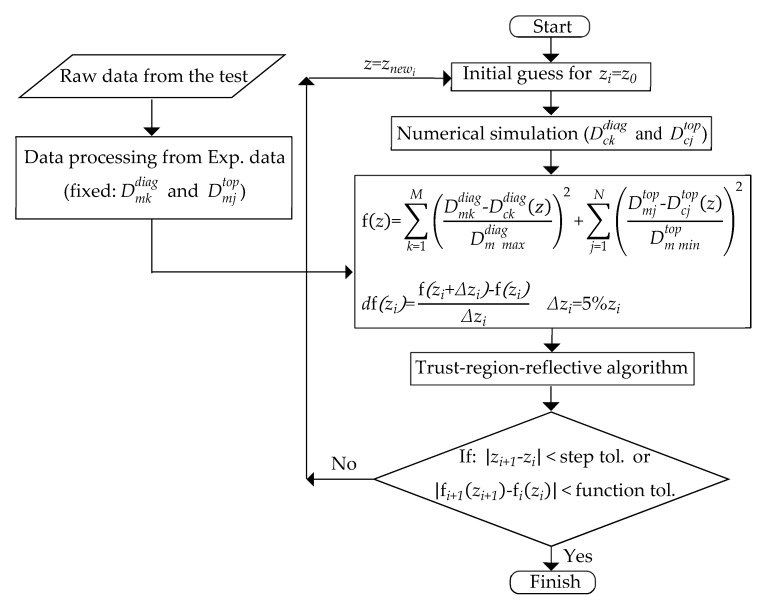
The designed algorithm for the identification procedure.

The identification procedure designed for the present application consists of different steps, as visualized in Figure 2. First, the initial guess of unknown parameters must be made, either from reference data or by arbitrary selection. However, since the trust-region-reflective algorithm must be supplied with upper and lower bounds for each parameter, the initial guess must be prescribed in the range of the predefined bounds. The selected ranges of nonlocal crystal plasticity parameters are $\tau_{0}\;\in \;\left[10,100\right]$ MPa, ${\hat{\tau }}_{sat}$ ∈ [100, 800] MPa, $c_{1}\;\in \;[0.01,0.08]$, and $p_{2}\;\in$ [2, 10].

Based on the initial guess of nonlocal crystal plasticity parameters, the primary numerical results developed by the finite element method will be obtained ($D_{ck}^{diag}$ and $D_{cj}^{top}$ in Equation (14)), and after processing the collected data acquired from the nanoindentation test $D_{mk}^{diag}$ and $D_{ck}^{diag}$ in Equation (14)), the discrepancy function f(*z*) can be computed, which is a scalar.

To increase the accuracy of the optimization procedure, the first derivative of the discrepancy function is computed by a typical central finite difference scheme, for which the increment has been set equal to 5% of the primary value of the corresponding nonlocal crystal plasticity parameter. Then, based on the selected algorithm, a new set of parameters and a new value of the discrepancy function will be found and utilized for the next iteration until achieving a minimum discrepancy function and finally the corresponding parameters by meeting one of the defined tolerances in the optimization. There are two tolerances that are considered as stopping criteria for the optimization algorithm: step tolerance and function tolerance. Step and function tolerances mean the difference between new sets of parameters and new discrepancy function at iteration (*i* + 1) and iteration (*i*), respectively. In this study, these tolerances were defined as 1 × 10^−4^.

### 3.2. Results and Model Verification

The optimization procedure was started with two different sets of initial nonlocal crystal plasticity parameters (using test data from the literature [74] and a set of arbitrary data to examine the robustness and convergence problems of the selected algorithm). Note that a set of arbitrary data in this study was randomly chosen. Due to the computational effort for the analysis of the numerical models, the identification problem was done only based on the results under 15 mN force, and then the uniqueness of the obtained optimized parameters was examined by applying other predefined forces (12.5, 17.5, and 20 mN).

Table 2 reports the initialization and the converged values of the nonlocal crystal plasticity parameters considered for ARMCO iron as well as the initial and final quantities of the discrepancy function, F. The final converged value of the discrepancy function verifies the selected algorithm since discrepancy functions from different initial sets approximately reached to the same quantity at the end of procedure. Obviously, the identified parameters based on the different initial data are close to each other, which justifies the uniqueness of the obtained nonlocal crystal plasticity parameters.

**Figure 3:**
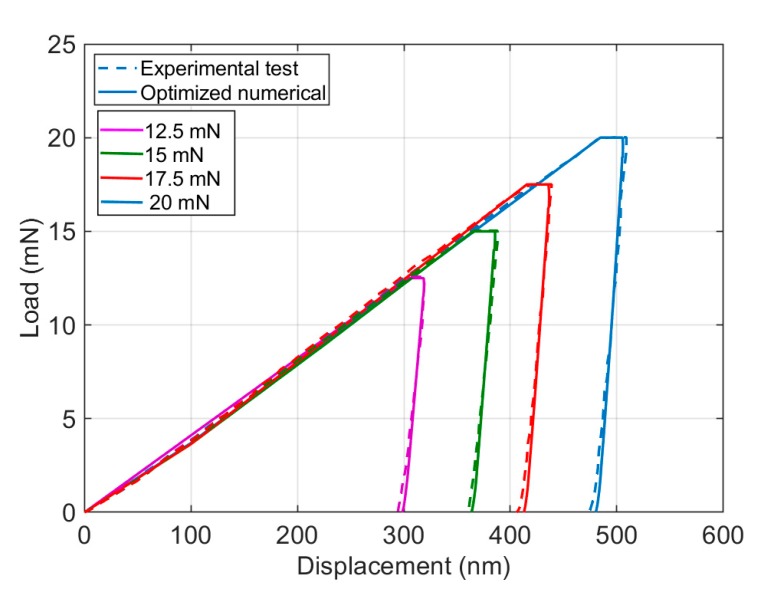
Comparison of load–displacement curves of experimental test and numerical model using identified parameters (initialized based on the literature) for different applied forces.

**Figure 4:**
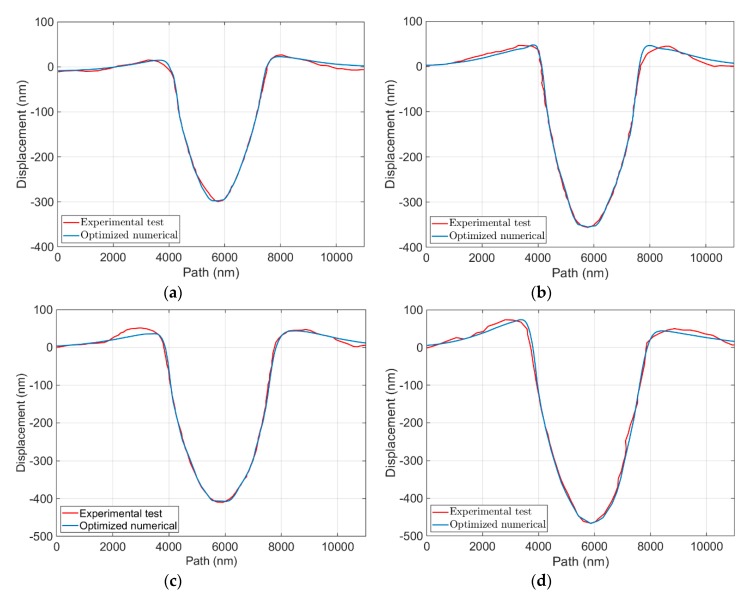
Comparison of load–displacement curves of the experimental test and numerical model using identified parameters (initialized based on the literature) for the forces of: (**a**) 12.5 mN; (**b**) 15 mN; (**c**) 17.5 mN; and (**d**) 20 mN.

In addition, the uniqueness of the identified parameters was also examined on load–displacement curves and on surface imprints by analyzing the numerical model based on the obtained parameters for different applied forces, as depicted in Figure 3 and Figure 4, in which the numerical model results for various applied forces are comparable with experimental output. Although, in Figure 4, there are small differences between experimental output and numerical results from maximum pile up to the edge of the surface, numerical results could follow the trend of the experimental output and capture the material behavior with a good agreement.

**Figure 5:**
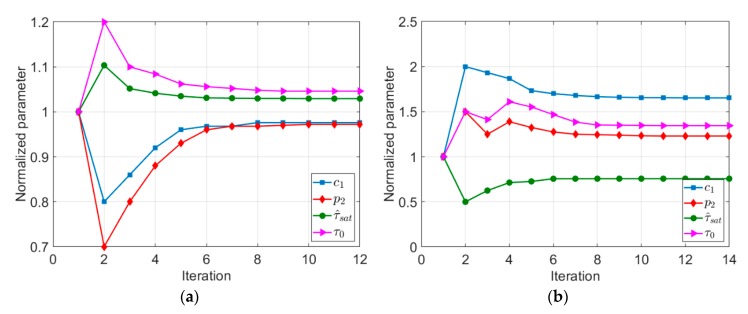
Trend of change in nonlocal crystal plasticity parameters based on: (**a**) the literature; and (**b**) arbitrary data.

The trend of changes in the considered parameters during the optimization procedure using different initial data (literature and arbitrary data) is shown in Figure 5. It is worth noting that the parameters visualized in Figure 5 were normalized by dividing them with their initial value.

**Figure 6:**
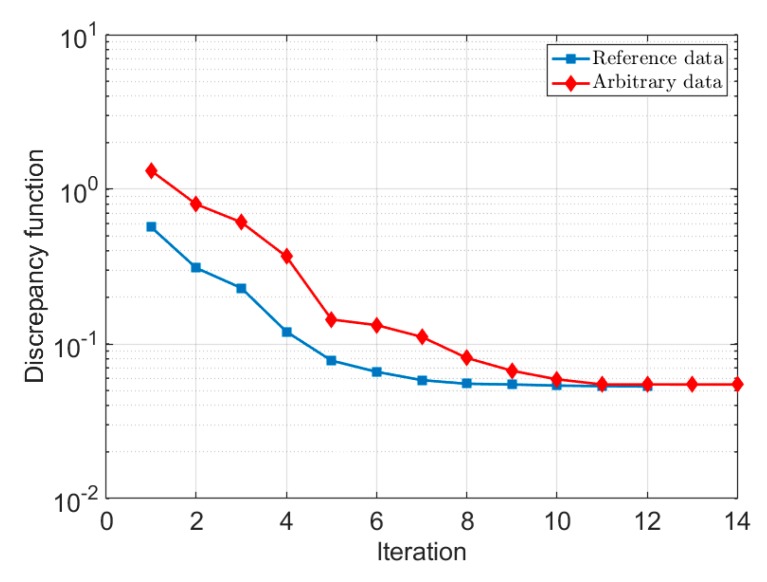
Trend of change in the discrepancy function during the optimization procedure.

The trend of change in the discrepancy functions at each iteration when using different initial sets is also shown in Figure 6. The same final values of the discrepancy function at the end of the inverse analysis under use of different sets of data justify that the identified parameters could reach the absolute minimum value of the discrepancy function in the optimization problem and not to the relative minimum, which is the reason of the uniqueness of the identified nonlocal crystal plasticity parameters shown for different applied loads.

## 4. Influence of Nonlocal Crystal Plasticity Parameters on the Nanoindentation Simulations

To evaluate the effect of different components on both the load–displacement curve and the surface imprint, the quantities of the preselected nonlocal crystal plasticity parameters (*c*_1_, *p*_2_, ${\hat{\tau }}_{sat}$, and $\tau_{0}$) are changed in a range. The obtained results in the following are for the applied force equal to 15 mN.

**Figure 7:**
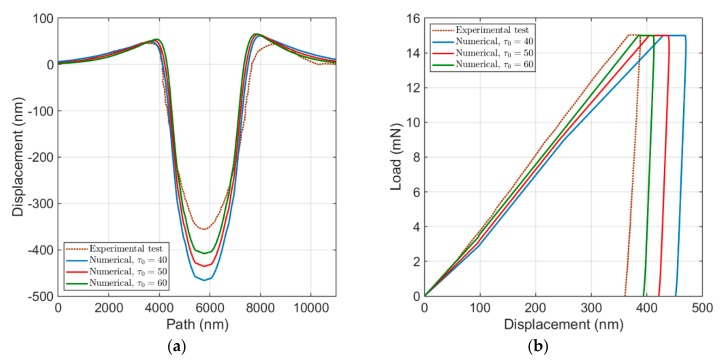
Influence of $\tau_{0}$: (**a**) the surface topology; and (**b**) the load–displacement curve.

Figure 7a compares the surface topology of the developed numerical model by variation in $\tau_{0}$, of which the most significant effect is on the penetration depth. By increasing $\tau_{0}$ and keeping the other parameters constant, the load–displacement curve shifts to the left side and tends to shift towards an agreement with the experimental test, as shown in Figure 7b. According to the described flow rule in Section 2.2, larger quantities of $\tau_{0}$ lead to a larger slip resistance and consequently to a reduction in plastic deformation. This effect becomes more visible by increase in $\tau_{0}$, which results in smaller level of displacement in load–displacement curve and lower level of penetration depth in surface topology. It also leads to a small increase of the maximum pile-up height. However, comparing to a larger influence of $\tau_{0}$ on the penetration depth, its influence on the maximum pile-up height is negligible.

**Figure 8:**
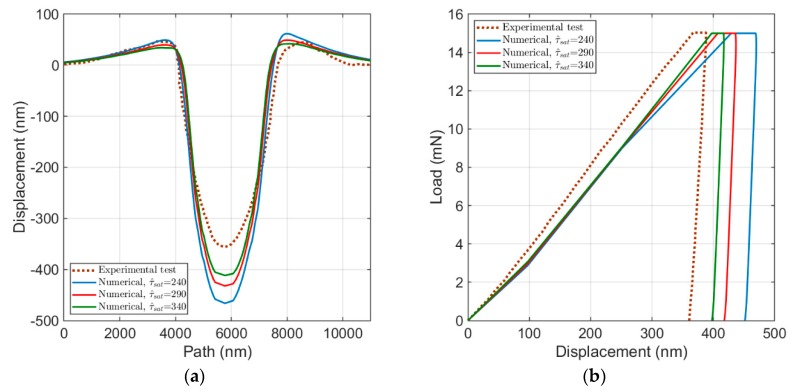
Influence of ${\hat{\tau }}_{sat}$: (**a**) the surface topology; and (**b**) the load–displacement curve.

Figure 8 represents the influence of ${\hat{\tau }}_{sat}$ on the load–displacement curve and on the surface imprints. By adopting different ${\hat{\tau }}_{sat}$ in numerical models, the maximum height (pile-up) reduces when ${\hat{\tau }}_{sat}$ increases, in addition to the reduction in the penetration depth (see Figure 8a). As visible in Figure 8b, similar to the effect of $\tau_{0}$, the load–displacement curves shift to the left side by increase in ${\hat{\tau }}_{sat}$, but the rate of change in displacement does not keep constant; the slope of the loading part roughly remains unchanged, and the width of the holding part of the load decreases. Because of the direct influence of the saturating critical resolved shear stress on the strain hardening law described in Equation (7), an increase in ${\hat{\tau }}_{sat}$ results in a reduction in the shear rate, which causes a lower level of stress and plastic deformations. This is also justifiable from the load–displacement curves and surface imprints.

**Figure 9:**
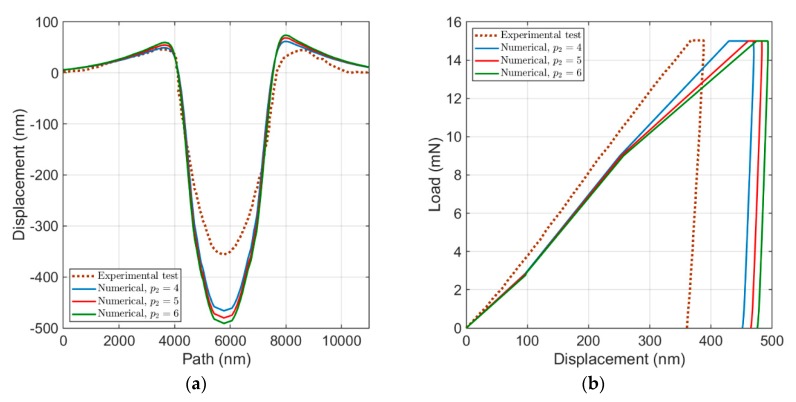
Influence of $p_{2}$: (**a**) the surface topology; and (**b**) the load–displacement curve.

By increasing the exponent of strain hardening, $p_{2}$, as shown in Figure 9a, both the maximum height and the penetration depth will increase. Unlike the effect of two former parameters ($\tau_{0}$ and ${\hat{\tau }}_{sat}$), the load–displacement curve moves to the right side and both the rate of change in displacement and width of holding part of the force decrease (see Figure 9b). The material response due to the increase in the exponent of strain hardening can also be explained by the flow rule. Since $p_{2}$ typically has quantities larger than 1, by its increase, τ^.α decreases in Equation (7), and hence the shear rate in the flow rule increases, which results in a higher level of stresses as well as in plastic deformations.

**Figure 10:**
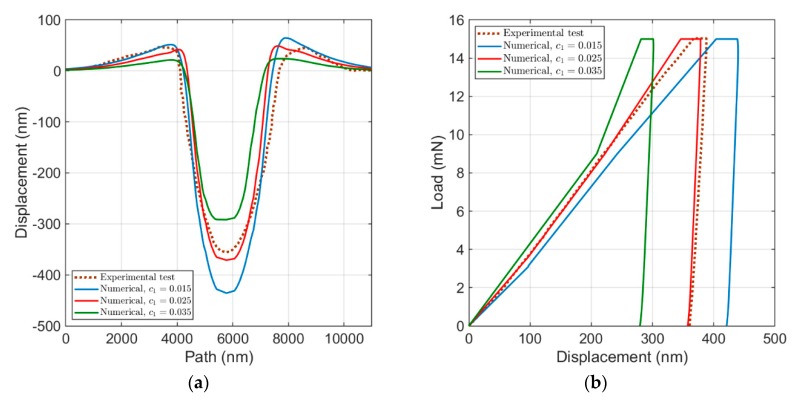
Influence of $c_{1}$: (**a**) the surface topology; and (**b**) the load–displacement curve.

The effect of $c_{1}$ on both the load–displacement curve and the surface topology is shown in Figure 10. Compared to the other parameters, the influence of $c_{1}$ on the material response is much more apparent. As visualized in Figure 10a, by an increase in $c_{1}$, both the maximum height and the penetration depth reduce. On the other hand, as shown in Figure 10b, an increase in $c_{1}$ leads to a shift of the load–displacement curves to the left side, an increase in the slope of the loading part, and a decrease in both the width of the holding part of the force and the corresponding displacement. The influence of the geometrical factor is on the additional hardening caused by GNDs (${\hat{\tau }}_{\alpha }^{GNDi}$). Thus, by increase in $c_{1}$, ${\hat{\tau }}_{\alpha }^{GNDi}$ also increases, which results in a reduction in the shear rate and, therefore, a decrease in plastic deformations in the slip system due to a lower level of stress.

Due to the complicated relationship between nonlocal crystal plasticity parameters and their complex influence on the material response under the nanoindentation test, a parametric study considering the simultaneous influence of a different combination of nonlocal crystal plasticity was conducted, in which the effect of only three pairs was described. In the following, the influence of three different combinations of nonlocal crystal plasticity parameters are studied on the load–displacement curve, penetration depth, and maximum depth.

**Figure 11:**
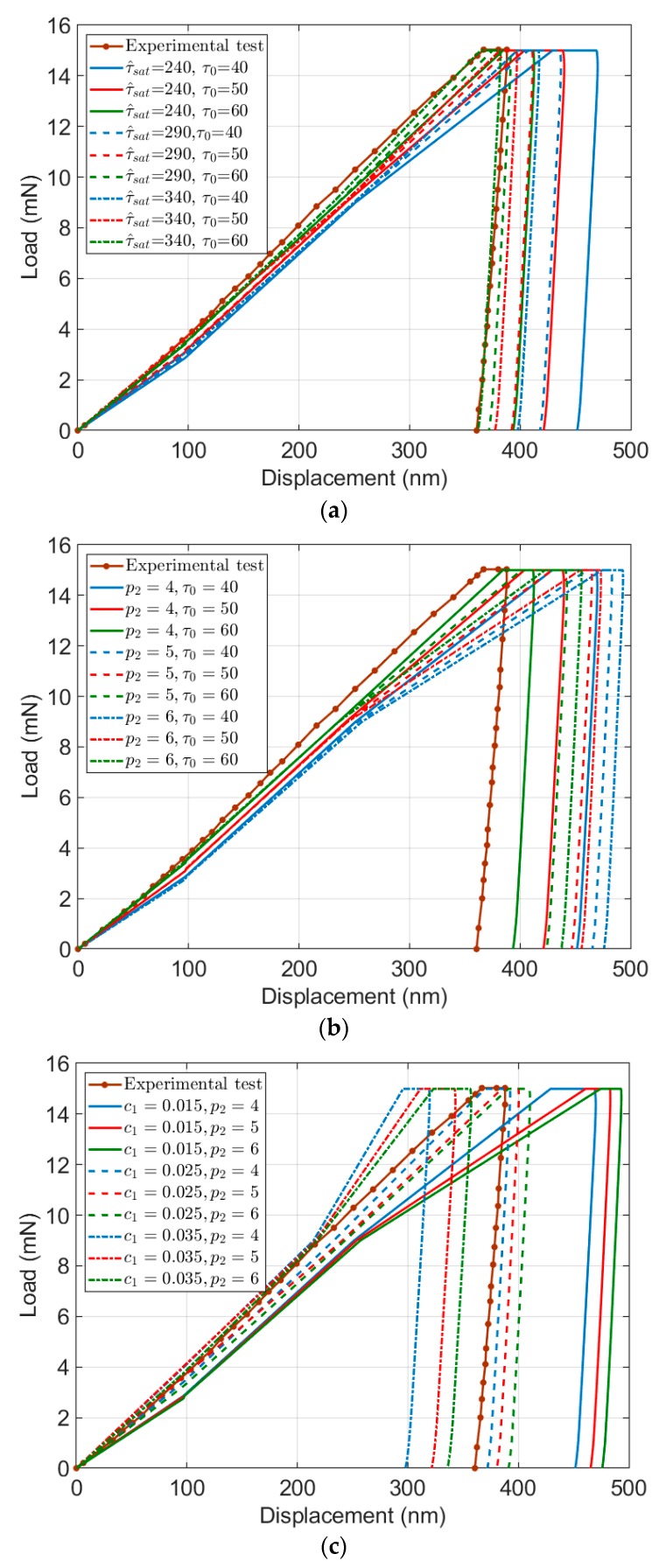
Load–displacement curve by simultaneous change in: (**a**) $\tau_{0}$ and ${\hat{\tau }}_{sat}$; (**b**) $\tau_{0}$ and $p_{2}$; and (**c**) $p_{2}$ and $c_{1}$.

As depicted in Figure 11a, by increase in both $\tau_{0}$ and ${\hat{\tau }}_{sat}$ values, the load–displacement curve shifts from the right to the left side, and the width of the holding part of the force will continuously decrease. Increasing $\tau_{0}$ leads to a decrease in displacement. At the same time, by increase in ${\hat{\tau }}_{sat}$, the rate of increase in displacement will also reduce. Furthermore, simultaneous increase in both $\tau_{0}$ and ${\hat{\tau }}_{sat}$ will rise the slope of the loading part. Overall, the range of changes in the load–displacement curves by variation in both $\tau_{0}$ and ${\hat{\tau }}_{sat}$, is very small, which is due to the counteracting role of these two parameters in the strain hardening law.

A simultaneous change in $\tau_{0}$ and $p_{2}$ results in the load–displacement curves shown in Figure 11b. By an increase in $\tau_{0}$, the load–displacement curve shifts from the right to the left side, but the width of the holding part of the force does not change. Furthermore, when $p_{2}$ goes up, the displacement increases, and the rate of this increase goes up for the higher $\tau_{0}$ quantities. Since the role of these two parameters on the strain hardening law is the opposite, very large or small values for both $\tau_{0}$ and $p_{2}$ will not significantly change the load–displacement curve.

The load–displacement curve by simultaneous change in $p_{2}$ and $c_{1}$ is shown in Figure 11c. By increase in both $p_{2}$ and $c_{1}$ quantities, the load–displacement curve generally shifts from the right to the left side. As is visible, an increase in $p_{2}$ leads to an increase in displacement, but at the same time, by an increase in $c_{1}$, the displacement will reduce. Furthermore, by an increase in the $c_{1}$ quantities, the slope of the loading part will increase. It is worth noting that the trend of changes in the holding part, by increase or decrease in both $p_{2}$ and $c_{1}$, is not clear.

The influence of both $\tau_{0}$ and ${\hat{\tau }}_{sat}$ on the penetration depth is studied in Figure 1a. In the case of simultaneous contributions of these two parameters, the penetration depth increases as they both increase and the minimum of it occurs when these two parameters have the lowest quantities. On the other hand, the penetration depth for the lowest value of $\tau_{0}$ ($\tau_{0}\;=\;40$ MPa) and the highest value of (${\hat{\tau }}_{sat}({\hat{\tau }}_{sat}=$ 340 MPa) is almost equal to the penetration depth for the highest value of $\tau_{0}$ ($\tau_{0}\;=\;60$ MPa) and the lowest value of (${\hat{\tau }}_{sat}({\hat{\tau }}_{sat}=$ 240 MPa). Moreover, the penetration depth remains almost unchanged in the linear variation between the two combinations of extreme values ($\tau_{0}\;=\;40$ MPa, ${\hat{\tau }}_{sat}=$ 340 MPa) and ($\tau_{0}\;=\;60$ MPa, ${\hat{\tau }}_{sat}=$ 240 MPa).

**Figure 12:**
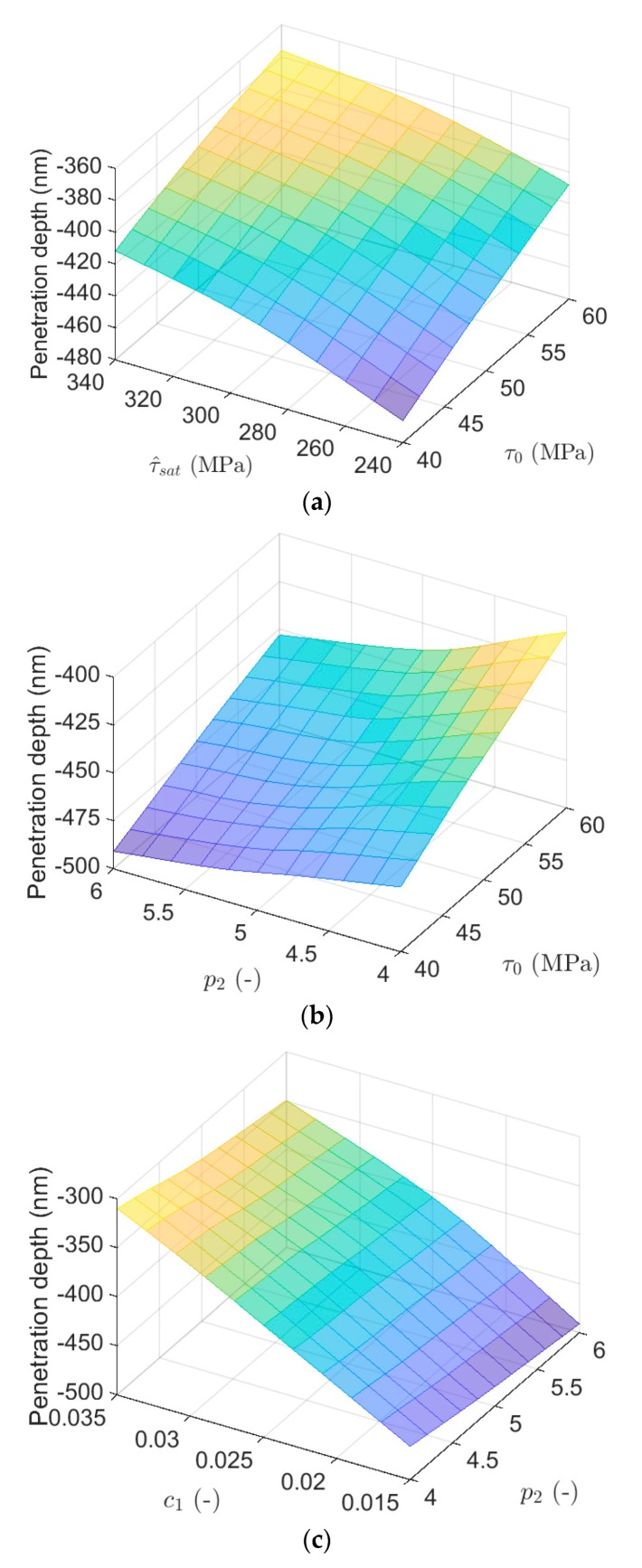
The influence on penetration depth of simultaneous change in: (**a**) $\tau_{0}$ and ${\hat{\tau }}_{sat}$; (**b**) $\tau_{0}$ and $p_{2}$; and (**c**) $p_{2}$ and $c_{1}$.

Figure 12b illustrates the variation of the penetration depth by combination of different quantities of $\tau_{0}$ and $p_{2}$. The maximum penetration depth occurs when $\tau_{0}$ is high but $p_{2}$ has a low value. Furthermore, the penetration depth for the lowest values of $\tau_{0}$ and $p_{2}$ ($\tau_{0}\;=\;40$ MPa and $p_{2}\;=\;4$) is almost equal to the penetration depth for their highest values ($\tau_{0}\;=\;60$ MPa and $p_{2}\;=\;6$). Here, the trend of change in the penetration depth with different $\tau_{0}$ is almost linear.

The effect of both $p_{2}$ and $c_{1}$ on the penetration depth is shown in Figure 12c. In the case of simultaneous contributions of these two parameters, the penetration depth increases as $p_{2}$ reduces and $c_{1}$ increases. It is worth noting that the penetration depth keeps approximately unchanged when only $p_{2}$ varies and it is more affected by variation in $c_{1}$. As is visible, by increase in $c_{1}$, the penetration depth increases significantly.

Due to complex relationship between parameters in the hardening law defined in Equations (6) and (7), it is difficult to estimate how these parameters interacts with each other during plastic deformation. The results shown in Figure 12 highlight the effect of different parameters combinations on the penetration depth and, where variation in $c_{1}$ significantly changes the penetration depth compared to the other parameters. It is also concluded that penetration depth increases by adopting higher level of geometrical factor, initial and saturation slip resistance, and lower level of strain hardening power. 

**Figure 13:**
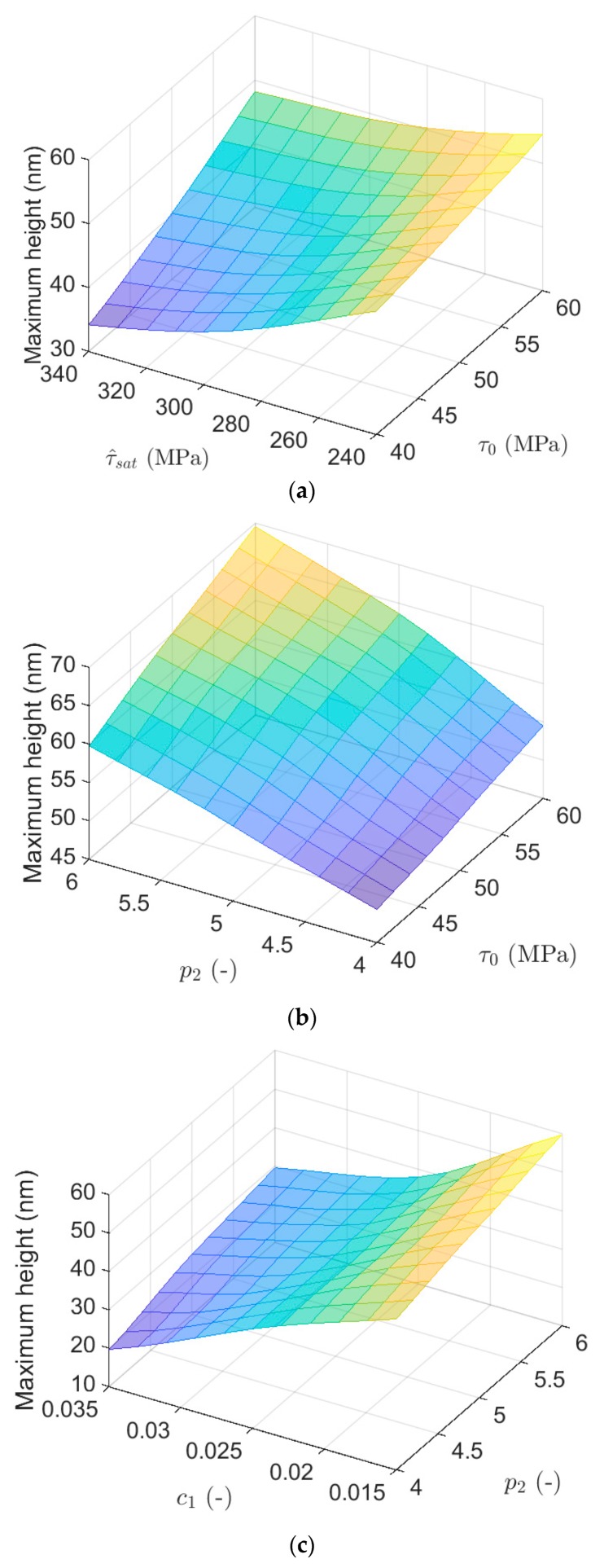
The influence on maximum height of simultaneous change in: (**a**) $\tau_{0}$ and ${\hat{\tau }}_{sat}$; (**b**) $\tau_{0}$ and $p_{2}$; and (**c**) $p_{2}$ and $c_{1}$.

Figure 13 shows variation of the maximum height considering combination of different parameters. As shown in Figure 13a, the maximum height has the maximum quantity when $\tau_{0}$ is high but ${\hat{\tau }}_{sat}$ has low value. Variation in maximum height is approximately linear by change in $\tau_{0}$. Furthermore, the maximum height for the lowest values of $\tau_{0}$ and ${\hat{\tau }}_{sat}$ ($\tau_{0}\;=\;40$ MPa and ${\hat{\tau }}_{sat}$ = 240 MPa) is almost equal to the maximum height for their highest values ($\tau_{0}\;=\;60$ MPa and ${\hat{\tau }}_{sat}$ = 340 MPa).

As is clear in Figure 13b, with an increase in $\tau_{0}$, the maximum height increases roughly linearly when $p_{2}$ has low value. In the case of simultaneous contributions of these two parameters, the maximum displacement increases as they both increase, and its minimum occurs when these two parameters have the lowest quantities. 

By a simultaneous change in both $p_{2}$ and $c_{1}$, Figure 13c visualizes that the maximum height has the highest quantity when $p_{2}$ is high but $c_{1}$ has a low value. Furthermore, the maximum height changes linearly with different $p_{2}$ but it varies completely nonlinearly with different $c_{1}$. 

The results presented in Figure 13 reflect higher variation for maximum height by changing the geometrical factor, as was also observed for the penetration depth. To increase the maximum height, it is needed to rise initial slip resistance and strain hardening power but reduce the saturation slip resistance and geometrical factor.

## 5. Conclusions

In the present study, a robust optimization scheme was developed and applied to experimental data to investigate the influence of nonlocal crystal plasticity parameters on the load–displacement and the surface topology of ARMCO iron under nanoindentation testing and to parameterize the predefined nonlocal crystal plasticity parameters by inverse analysis.

The identification process started with different initial guesses for nonlocal crystal plasticity parameters, which were chosen from the literature and arbitrarily. The results (load–displacement curves and surface topologies) show a converged solution at the end of the optimization procedure reaching the minimum discrepancy function. The identification algorithm was done by considering the load–displacement curve and the surface topology for an applied force of 15 mN. Then, to examine the uniqueness of the identified parameters, the load–displacement curve and the surface imprint extracted from the experimental test were compared with the numerical analyses for various applied forces (12.5, 17.5, and 20 mN).

The sensitivity analyses were done in the numerical model by varying the preselected nonlocal crystal plasticity parameters (*c*_1_, *p*_2_, ${\hat{\tau }}_{sat}$ and $\tau_{0}$). In surface imprints, maximum height was mostly unaffected by $\tau_{0}$ and $p_{2}$ but reduced by increasing ${\hat{\tau }}_{sat}$ and $c_{1}$. When *c*_1_, ${\hat{\tau }}_{sat}$ and $\tau_{0}$ increased, the penetration depth reduced while $p_{2}$ had an inverse effect such that its increase resulted in deeper penetration depth. The results highlight that the geometrical factor, $c_{1}$, has the most significant influence on both load–displacement curves and surface imprints in comparison with the other parameters. 

## Figures and Tables

**Table 1:** Crystal plasticity parameters of ARMCO iron.

Parameter	Notation	Value
Elastic constant	$C_{11}$ (GPa)	231
Elastic constant	$C_{12}$ (GPa)	134.7
Elastic constant	$C_{44}$ (GPa)	116.4
Initial slip resistance	$\tau_{0}$ (MPa)	50
Saturation slip resistance	${\hat{\tau }}_{sat}$ (MPa)	290
Inverse of strain rate sensitivity	$p_{1}$ (–)	26.7
Exponent of strain hardening	$p_{2}$ (–)	5.0
Initial hardening rate	$h_{0}$ (MPa)	961
Geometrical factor	$C_{1}$ (–)	0.025
Average dislocation pile-up size	L (nm)	1
Cross hardening coefficient	$\chi_{\alpha \beta }^{GND}$(–)	1

**Table 2:** Optimized nonlocal crystal plasticity parameters using nanoindentation test.

Parameter	Initialization Value (Literature)	Optimized Value	Parameter	Initialization Value (Arbitrary)	Optimized Value
$c_{1}$ (–)	0.025	0.0244	$c_{1}$ (–)	0.015	0.0245
$p_{2}$ (–)	5	4.86	$p_{2}$ (–)	4	4.92
${\hat{\tau }}_{sat}$ (MPa)	290	298.5	${\hat{\tau }}_{sat}$ (MPa)	400	302.8
$\tau_{0}$ (MPa)	50	52.3	$\tau_{0}$ (MPa)	40	53.8
f(z) (–)	0.57	0.05312	f(z) (–)	1.321	0.05468
